# C3-Liposome Delivery of MUC1 Peptide and TLR Agonists Enhances Adaptive Immunity and Results in Sex-Based Tumor Growth Differences

**DOI:** 10.3390/pharmaceutics17040468

**Published:** 2025-04-03

**Authors:** Shahab Soltani, Ameneh Arabi, Kristine Mann, Austin Hess, Holly A. Martinson, Max Kullberg

**Affiliations:** 1 WWAMI School of Medical Education, University of Alaska Anchorage, 3211 Providence Drive, Anchorage, AK 99508, USA; ssoltani@hivresearch.org (S.S.); ahess10@alaska.edu (A.H.); hamartinson@alaska.edu (H.A.M.); 2 Henry Jackson Foundation for the Advancement of Military Medicine, Walter Reed Army Institute of Research, Silver Spring, MD 20910, USA; 3 Department of Biological Sciences, University of Alaska Anchorage, Anchorage, AK 99508, USA; kmann1@alaska.edu

**Keywords:** antigen-presenting cell, vaccine, cancer immunotherapy, complement C3, liposome, nanoparticle, targeted delivery, adjuvants, toll-like receptor, mucin-1, Lewis lung carcinoma

## Abstract

**Background**: Mucin-1 (MUC1) is a glycoprotein that is hypoglycosylated and overexpressed in most adenocarcinomas, making it a promising target for cancer vaccines. Our group previously demonstrated that C3 (OPSS)-liposomes enhance antigen uptake by antigen-presenting cells (APCs) via the complement C3 pathway and, when combined with toll-like receptor (TLR) agonists, reduce tumor growth in murine cancer models. **Methods**: In the present study, we evaluate the immunogenicity of MUC1 peptide vaccines encapsulated in C3-liposomes, with and without TLR agonists, using MUC1-tolerant transgenic mice challenged with Lewis lung carcinoma (LLC.MUC1) cells. To assess vaccine effectiveness, tumor volumes were measured, and flow cytometry and ELISA and ELISPOT assays were used to assess the immune response. **Results**: Both male and female C57BL/6 transgenic mice vaccinated with MUC1 C3-liposomes developed significantly smaller tumors than those vaccinated with free MUC1 peptide or PBS. Notably, a sex-dependent response emerged in mice vaccinated with MUC1 C3-liposomes with TLR agonists (TLR4, TLR7/8, and TLR9); male mice exhibited greater tumor suppression than females. Flow cytometry analysis revealed that female mice had significantly higher levels of CD11b^+^, LY6C^+^, and LY6G^+^ MDSC cells, suggesting a potential mechanism for the sex difference. Additionally, MUC1 C3-liposome vaccination elicited robust adaptive immune responses, including significantly higher levels of IFN-γ-producing T cells and MUC1-specific IgG antibodies compared to non-encapsulated MUC1 or TLR adjuvant-only formulations. **Conclusions**: These findings underscore the potential of C3-liposome-based antigen vaccines to enhance anti-tumor immunity and highlight the impact of sex differences in vaccine efficacy.

## 1. Introduction

Cancer vaccines are intended to establish a specific immune response against tumor antigens, leading to enhanced antibody and T-cell recognition of cancer cells. Among recognized tumor antigens, mucin-1 (MUC1) remains one of the most prevalent targets [1,2]. MUC1 was identified by Dr. Olivera Finn in 1989 and is aberrantly expressed in a hypoglycosylated form in various epithelial adenocarcinomas, such as breast, lung, liver, and colorectal cancer [3]. Almost all epithelial cells express MUC1 at low levels on their apical surfaces, where the protein functions to form a protective barrier that prevents colonization by pathogens and aids in the lubrication of epithelia [2,4]. MUC1 is a transmembrane protein that has an extracellular region characterized by the presence of a variable number of tandem repeats (VNTRs) containing 20 amino acids (HGVTSAPDTRPAPGSTAPPA), a transmembrane domain, and a cytoplasmic tail domain. On healthy epithelial cells, each VNTR region contains five O-glycosylation sites that are heavily glycosylated [5].

The prevalence of overexpressed and hypoglycosylated MUC1 in cancer cells and its accessibility on the cell surface make MUC1 a possible target for cancer vaccines. Several vaccines have been developed based on MUC1 antigens over the past few decades, including subunit, DNA, and viral vaccines [5]. These MUC1 vaccine studies have demonstrated that the MUC1 peptide is highly immunogenic and that vaccination provides immune protection against transplantable or spontaneous MUC1-expressing tumors [6,7]. Vaccines that include adjuvants generally induce a more robust immune response than vaccines without adjuvant [8]. Adjuvants that are commonly used to boost the immune response of cancer vaccines include Bacillus Calmette–Guerin (BCG), stable emulsion adjuvant system 2 (SB-AS2), maltose-binding protein (MBP), and toll-like receptor (TLR) agonists [9,10]. For this study, we utilized TLR agonists as adjuvants based on several established vaccines that showed promising results using a combination of MUC1 peptide and TLR agonists [9,11,12,13].

Delivery strategies for peptide vaccines include the use of nanoparticles, self-assembling peptides, electroporation, viral vectors, and gene gun vaccine delivery [14]. Nanoparticle platforms can address pharmacokinetic limitations such as short half-life, poor bioavailability, and poor solubility, and include liposomes, polymeric nanoparticles, gold nanoparticles, and virus nanoparticles [15]. One way to deliver antigens is via liposomes, a versatile delivery system that can be modified by changing the lipid composition, charge, size, and surface properties [16,17]. Antigens can be delivered more effectively and specifically to APCs using targeted liposome nanoparticles [18]. Among the liposomal-targeted delivery systems are mannose, Fc-targeted, CD11c-targeted, and cationic liposomes [19,20]. Our lab has developed C3-liposomes that contain an exposed ortho pyridyl disulfide (OPSS) group, which binds to endogenous complement C3, resulting in the targeted delivery of antigens directly to APCs through the C3b receptor [16,17]. In addition to antigens, the C3-liposome delivery system can easily incorporate and deliver vaccine adjuvants like TLR agonists.

In this study, we utilized C3-liposomes to deliver TLR agonists and a MUC1 100mer peptide developed by Dr. Olivera Finn, with the goal of enhancing the immunogenicity of the MUC1 100mer peptide. Based on promising results from our previous C3-liposome MUC1 vaccine study [21], we chose to encapsulate TLR4, TLR7/8, and TLR9 agonists with the MUC1 100mer peptide inside C3-liposomes. The vaccine was tested prophylactically in MUC1 transgenic mice (MUC1.Tg), which exhibit tolerance to engrafted MUC1 transfected Lewis lung carcinoma (LLC) murine cells. After vaccination, MUC1.Tg mice were inoculated with LLC cells as a tumor challenge model to assess the induction of an immunogenic and anti-tumor response. Our results demonstrated that the MUC1 C3-liposome vaccine could effectively hinder tumor growth and induce robust antibody and T-cell immune responses against MUC1-positive tumor cells.

## 2. Materials and Methods

### 2.1. Reagents

The human 100mer MUC1 was synthesized by Celtek peptides (Franklin, TN, USA) and based on the design by Dr. Olivera Finn’s laboratory [7]. For liposome production, lipids 1,2-dipalmitoyl-sn-glycero-3-phosphocholine (DPPC), 1,2-distearoyl-sn-glycero-3-phosphocholine (DSPC), 1,2-distearoyl-sn-glycero-3-phosphoethanolamine-N-[poly(ethylene glycol)-2000] (DSPE-PEG(2000)), and 1,2-distearoyl-sn-glycero-3-phosphoethanolamine-N-[PDP-poly(ethylene glycol)-2000] (DSPE-PEG(2000)-PDP) were obtained from Avanti Polar Lipids (Alabaster, AL, USA). CpG 1826 was purchased from TriLink (San Diego, CA, USA). R848 and MPLA were purchased from InvivoGen (San Diego, CA, USA). CL-4B Sepharose gel for size exclusion chromatography was obtained from Sigma-Aldrich (St. Louis, MO, USA). TMB ELISA substrate, goat anti-mouse IgG H&L, streptavidin–horseradish peroxidase (HRP), and mouse anti-human CD227 antibody (clone C595), recognizing tandem repeat domain within the extracellular portion of the MUC1 protein, were purchased from Bio-Rad (Hercules, CA, USA). ChonBlock blocking/sample dilution ELISA and ChonBlock detection antibody dilution buffers were purchased from Chondrex (Woodinville, WA, USA). Flow cytometry antibodies, FITC anti-mouse CD45 (30-F11), PerCP/Cy5.5 anti-mouse Ly6G (1A8), PE/DAZZLE anti-mouse CD19 (6D5), PE/Cy7 anti-mouse CD11c (N418), APC anti-mouse CD3 (17A2), Alexa Fluor 700 anti-mouse/human CD11b (M1/70), APC/Cy7 anti-mouse CD8b (YTS156.7.7), BV510 anti-mouse Ly6C (HK1.4), BV605 anti-mouse MHCII (M5/114.15.2), BV650 anti-mouse F4/80 (BM8), and BV785 anti-mouse CD4 (GK1.5) were obtained from BioLegend (San Diego, CA, USA), with the clone of each antibody shown in parentheses. All other reagents were purchased from Fisher Scientific (Waltham, MA, USA). The EnzyChrom™ Alanine Transaminase Assay Kit and the EnzyChrom™ Aspartate Transaminase Assay Kit for liver toxicity assay were purchased from BioAssay systems (Hayward, CA, USA).

### 2.2. Tumor Cell Line

The Lewis lung carcinoma (LLC) cell line was stably transfected with human MUC1 cDNA and kindly provided by Dr. Olivera Finn (University of Pittsburgh, Pittsburgh, PA, USA) [22]. Tumor cells (LLC.MUC1) were cultured in Dulbecco’s modified Eagle medium with fetal bovine serum and penicillin/streptomycin [DMEM (89%), heat-inactivated FBS (10%), pen/strep (1%)], and incubated at 37 °C in 5% CO_2_.

### 2.3. Liposome Preparation

Liposomes were prepared by film hydration–extrusion as described previously [16,17]. To create OPSS liposomes, the lipids DPPC/DSPC/DSPE-PEG-2000-PDP/DSPE-PEG-2000 were mixed in a ratio of 76:18:3:3. Briefly, liposomes were prepared by dissolving lipids in chloroform, drying them under nitrogen for one hour, and rehydrating them with 0.7 mL of MUC1 (1 mg/mL DI water). For control C3-liposomes that did not contain MUC1 antigen, the liposomes were rehydrated with 0.7 mL deionized water. The liposomes were extruded nine times at 47 °C through an Avanti Mini Extruder (Avanti Polar Lipids). For liposomes containing TLR agonists, 60 μL of MPLA (1 mg/mL) (TLR4 agonist) was added to the lipids before drying under nitrogen, and a mixture of CpG 1826 (4.5 mg) (TLR9 agonist), R848 (1 mg) (TLR 7/8 agonist), and the 100mer MUC1 (0.7 mg), if present, were added to the DI water in a total volume of 0.7 mL for rehydration. Liposomes were separated from non-encapsulated material using a CL-4B Sepharose column hydrated with PBS (pH 7.4). Liposome size was determined with a Malvern Zetasizer Nano-S (Malvern Instruments, Malvern, UK). HPLC was used to match encapsulated MUC1 and R848 concentrations between the respective groups using HPLC methods previously described [21].

### 2.4. Mouse Model, Vaccination Groups, and Immunization

C57BL/6-Tg(MUC1)79.24Gend/J (MUC1.Tg mice) transgenic mice and C57BL/6J mice were purchased from the Jackson Laboratory (Bar Harbor, ME, USA) and housed in the University of Alaska Anchorage (UAA) vivarium. A mouse colony was maintained by breeding wild-type C57BL/6 females with heterozygous MUC1.Tg males. Hemizygous MUC1.Tg mice were identified by PCR amplification of tail DNA with MUC1 primers to confirm the presence of the MUC1 sequence. Five groups of mice, each including four male and four female MUC1.Tg.mice (8–12 weeks old), were vaccinated with the following vaccine formulations: group 1: MUC1 with triple TLR agonists TLR4, TLR7/8, and TLR9 encapsulated in C3-liposomes (MUC1 3Adj C3-liposomes); group 2: MUC1 encapsulated in C3 liposomes (MUC1 C3-liposomes); group 3: free MUC1 peptide; group 4: triple TLR agonists TLR4, TLR7/8, and TLR9 encapsulated in C3-liposomes (3Adj C3-liposome); group 5: PBS. On day one, the vaccine or PBS was subcutaneously injected into the left flank of the mouse, and a booster vaccination was administered in the same flank seven days later. Fourteen days post-initial vaccination, tumor cells were inoculated into the right flank of the mice. MUC1-transfected LLC cells were isolated and resuspended at a concentration of 5 × 10^4^ cells in 50 μL of PBS, then injected into the right flank of the mice under anesthesia (isoflurane). Tumor measurements were taken once the tumors were palpable and measured every other day thereafter using a digital caliper. Tumor volumes were calculated as mm^3^ [(4/3)π(length ×width × minimum)/8]. The mice were euthanized 28 days after tumor injection; spleens, tumors, and blood were collected and analyzed by flow cytometry, ELISpot, ELISA, and hepatotoxicity. All experiments were approved by the UAA Institutional Animal Use and Care Committee (IACUC), and the mice were monitored daily for signs of distress.

### 2.5. Flow Cytometry Analysis of Spleen Cells

Splenocytes were collected from mice, digested with collagenase, and analyzed by flow cytometry as previously described [17]. Cells resuspended in FACS buffer were stained with fluorescent antibodies to CD45, Ly6G, CD19, CD11c, CD3, CD11b, CD8b, Ly6C, MHCII, F4/80, and CD4 and analyzed using a Beckman Coulter CytoFLEX flow cytometer with CytExpert software version 2.0.0.153 (Beckman Coulter, Brea, CA, USA).

### 2.6. ELISA Assay

The ELISA assay was performed to detect MUC1-specific IgG antibodies in serum samples of all mice at 6 weeks post-vaccination. Ninety-six-well flat-bottom plates (Nunc MaxiSorp) were coated with 100 μL of 100mer MUC1 peptide (1 μg/mL) incubated at 4 °C overnight. Serum samples were diluted 1:200 in CHONBLOCK solution (Chondrex, WA, USA) and added to the wells. The manufacturer’s protocol was followed for the rest of the ELISA assay. Mouse anti-human CD227 antibodies (BioRad, Hercules, CA, USA) were used to generate a standard curve, while non-coated wells served as the negative control. The absorbance was measured using an ELISA reader from Molecular Devices (Spectramax, iD3) at 650 nm.

### 2.7. ELISPOT Assay

Quantification of IFN-γ-producing T cells against MUC1 antigen in vaccinated mice was assessed with a murine IFN-γ single-color enzymatic ELISpot kit (ImmunoSpot^®^test kit, Cleveland, OH, USA), using the procedure previously described (21). Briefly, spleens were digested with 1 mL of collagenase (1 mg/mL) and counted using a Cell Drop Automated Cell Counter (DeNovix). Each well was seeded with 100 μL of spleen cells (4 × 10^5^ cells) and 6 μL of 100mer MUC1 (1 mg/mL) mixed with 94 μL CTL-TestTM medium. Following 24 h of incubation at 37 °C and 9% CO_2_, the ImmunoSpot ELISpot kit was used to detect the number of T cell clones producing IFN-γ. A CTL ImunoSpot S6 Micro analyzer (ImmunoSpot, OH, USA) was used to read and analyze the ELISpot plate following overnight drying at room temperature.

### 2.8. Liver Toxicity Assays

To determine liver toxicity, alanine transaminase (ALT) and aspartate aminotransferase (AST) were measured for liver toxicity with an EnzyChromTM Alanine/Aspartate Transaminase Assay Kit (BioAssay Systems, Hayward, CA, USA), following the manufacturer’s instructions. The plate was incubated at room temperature for 10 min before the absorbance was measured using an absorbance reader from Molecular Devices (Spectramax, iD3) at 340 nm.

### 2.9. Statistical Analysis

Statistical analysis was performed using GraphPad Prism 9 software. Student’s unpaired *t*-test and the Mann–Whitney U test were performed to compare the means of two independent or unrelated groups. All analyses were considered statistically significant when the *p*-value was 0.05 or less. Asterisks indicate statistically significant differences (**, *p* = 0.01; *, *p* = 0.05; n.s., not significant).

## 3. Results

### 3.1. MUC1 C3-Liposomes Slow Tumor Growth in MUC1.Tg Mice

**Figure 1:**
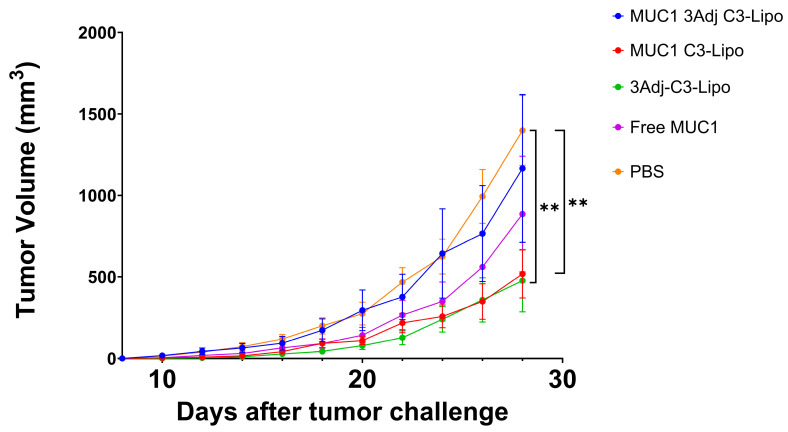
Mice vaccinated with MUC1 C3-liposomes had reduced tumor growth. Following vaccination on days 1 and 7, MUC.Tg mice were challenged with LLC.MUC1 tumor cells injected subcutaneously (s.c.) on day 14 into the right flank. Tumor volumes were measured every other day from day 10 post-inoculation until day 28. Mice vaccinated with MUC1 C3-liposomes (MUC1 C3-Lipo) and 3Adj C3-liposomes (3Adj C3-Lipo) developed significantly smaller tumors compared to PBS control mice (*p* = 0.006, 0.004 respectively). No significant differences in tumor growth were observed in mice vaccinated with MUC1 3Adj C3-liposomes (MUC1 3Adj C3-Lipo) or with free MUC1 compared to the PBS control group. Data are expressed as mean ± standard error (N = 8). ** *p*-value < 0.01.

MUC1.Tg mice were vaccinated on days one and seven with one of the following formulations: MUC1 C3-liposomes containing TLR adjuvants (MUC1 3Adj C3-liposomes), MUC1 C3-liposomes with no TLR adjuvants (MUC1 C3-liposomes), C3-liposomes with TLR adjuvants but no MUC1 (3Adj C3-liposomes), free MUC1 peptide, or PBS control. To determine whether C3-liposome delivery of MUC1 peptide with TLR agonists slows tumor growth, mice were challenged with MUC1-transfected Lewis lung carcinoma (LLC) tumor cells fourteen days following vaccination, and the mice were monitored for tumor growth. Mice vaccinated with MUC1 C3-liposomes developed significantly smaller tumors than PBS controls (Figure 1). This contrasts with free MUC1, which did not result in significantly different tumor sizes compared to PBS controls. These results suggest that C3-liposome delivery of the MUC1 peptide may enhance vaccine efficiency and efficacy, resulting in slower tumor growth (Figure 1). P-values for all significant data in the paper can be found in the Supplementary Materials (Table S1).

**Figure 2:**
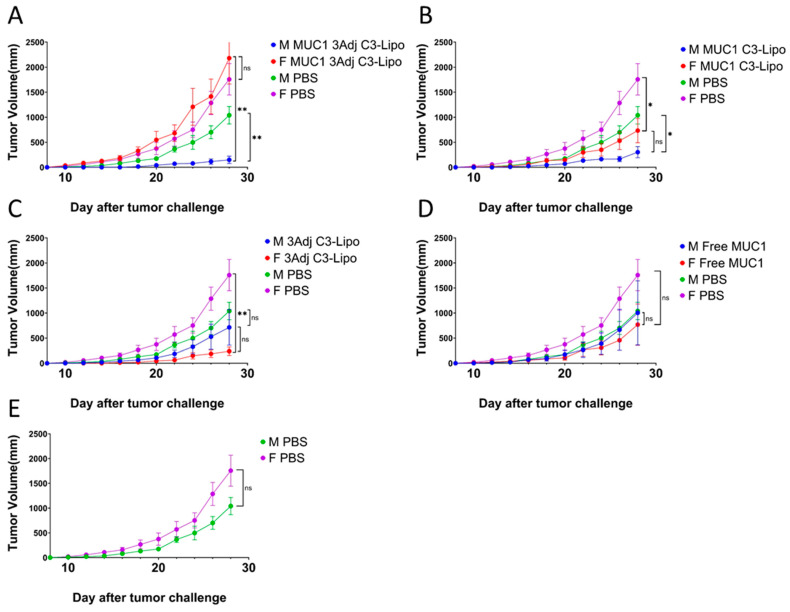
Sex differences in tumor growth after vaccination and tumor challenge. To assess sex differences in tumor growth, individual graphs show tumor growth in male and female mice for each treatment compared to PBS. (**A**) Female mice vaccinated with combined MUC1 and TLR agonists in C3-liposomes had significantly greater tumor growth compared to male mice. Male mice vaccinated with MUC1 3Adj C3-liposome developed significantly smaller tumors compared to control male PBS mice. (**B**) Male and female mice vaccinated with MUC1 C3-liposomes developed smaller tumors compared to control mice. (**C**) Female mice vaccinated with 3Adj C3-liposomes developed significantly smaller tumors compared to control PBS female mice. (**D**,**E**) No significant difference in tumor volume was observed between male and female mice that received either free MUC1 or PBS. Data are expressed as mean ± standard error (N = 4). (* *p*-value < 0.05, ** *p*-value < 0.01, ns (not significant)).

To further bolster the immune response, C3-liposomes were formulated with encapsulated TLR adjuvants (R848, CpG, and MPLA). When mice were vaccinated with C3-liposomes containing TLR agonists and no MUC1, tumor growth was significantly reduced compared to PBS controls. However, when C3-liposomes were formulated with MUC1 and TLR agonists, the combination seemed detrimental, as it abolished the significant reduction in tumor growth seen in MUC1 C3-liposome-vaccinated mice (Figure 1). This result did not align with our initial hypothesis that combined delivery of MUC1 peptide and TLR agonists in C3-liposomes would synergistically enhance the immune response against cancer. It was not until the groups were separated by sex that it was apparent that there were significant sex-based differences in tumor growth when both MUC1 antigen and TLR agonists were combined into a single set of C3-liposomes (Figure 2).

### 3.2. C3-Liposome Delivery of MUC1 Antigen and TLR Agonists Resulted in Sex Differences in Tumor Growth

To further evaluate this unexpected outcome, vaccine groups were divided based on sex and reanalyzed (Figure 2). We observed significant sex differences in tumor growth in the group that received the combined vaccine of MUC1 and TLR agonists in C3-liposomes (Figure 2A). Female mice had significantly larger tumors compared to male mice receiving the same vaccine (*p* = 0.003) (Figure 2A). Moreover, male mice from this vaccine group developed significantly smaller tumors than male PBS mice (*p* = 0.008) (Figure 2A). These results suggest sex differences in response to vaccination when TLR agonists and MUC1 peptides are delivered in combination in C3-liposomes. We observed no sex differences in tumor volume in the MUC1 C3-liposome group that did not contain TLR agonists, with both sexes having significantly smaller tumors than PBS mice (female mice: *p* = 0.04, male mice: *p* = 0.01) (Figure 2B). Immunized female mice with TLR agonists in C3-liposomes that did not contain MUC1 antigen developed significantly smaller tumors than control PBS female mice (*p* = 0.003), a result that we did not see in male mice (Figure 2C). Male mice required the combination of MUC1 antigen and TLR agonists to establish significantly reduced tumor growth compared to PBS mice. These results indicate that male and female mice have differences in immune response when antigen is combined with TLR agonists in C3-liposomes, resulting in tumor growth patterns that are not immediately straightforward to predict. The adaptive immune response was therefore analyzed to determine whether differences in T cell and antibody response were driving tumor growth differences.

### 3.3. MUC1-Specific T-Cell Response in Mice Vaccinated with MUC1 C3-Liposome Formulations

**Figure 3:**
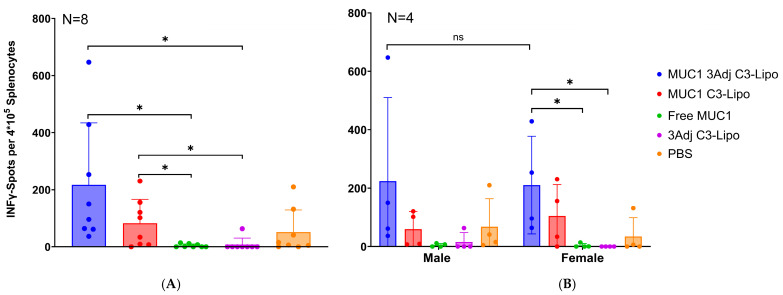
MUC1 C3-liposome formulations induce MUC1-specific T-cell responses in vaccinated mice. Spleen cells from vaccinated mice were stimulated with MUC1 antigen and analyzed for MUC1-specific T-cell activation using an ELISpot assay. (**A**) MUC1 3Adj C3-liposome-vaccinated and MUC1 C3-liposome-vaccinated mice had significantly higher levels of IFN-γ-producing T cells compared to mice vaccinated with free MUC1 or 3Adj C3-liposomes (N = 8). (**B**) Female mice vaccinated with MUC1 3Adj C3-liposomes had significantly more IFN-γ-producing T cells than female mice vaccinated with free MUC1 (*p* = 0.04) or 3Adj C3-liposomes (*p* = 0.04) (N = 4). Data are expressed as mean ± standard error. * *p*-value < 0.05, ns (not significant).

To assess the development of a MUC1-specific T-cell response in vaccinated MUC1.Tg mice, isolated spleen cells were stimulated with MUC1 peptide in an ELISpot assay. Mice vaccinated with MUC1 3Adj C3-liposome had significantly more IFN-γ producing T cells compared to mice vaccinated with free MUC1 (*p* = 0.01) or 3Adj C3-liposomes (*p* = 0.01) (Figure 3A). In addition, mice vaccinated with MUC1 C3-liposomes had significantly more IFN-γ-producing T cells than mice vaccinated with either free MUC1 (*p* = 0.02) or 3Adj C3-liposomes (*p* = 0.02) (Figure 3A). These results indicate that C3-liposome encapsulation of MUC1 antigen and TLR agonists significantly enhances a MUC1-specific T-cell response.

No significant differences in MUC1-specific T-cell responses were observed between male and female mice within each vaccine group (Figure 3B). Similarly, the number of IFN-γ-producing T cells did not differ among vaccinated male mice (Figure 3B). However, female mice vaccinated with MUC1 3Adj C3-liposomes exhibited significantly more IFN-γ-producing T cells than those receiving free MUC1 (*p* = 0.04) or 3Adj C3-liposomes (*p* = 0.04) (Figure 3B). This robust T-cell response in females vaccinated with MUC1 3Adj C3-liposome was notable, as these mice developed significantly larger tumors, suggesting the presence of systemic or localized tumor-induced immune suppression.

### 3.4. C3-Liposomes Enhance a MUC1-Specific IgG Antibody Response

**Figure 4:**
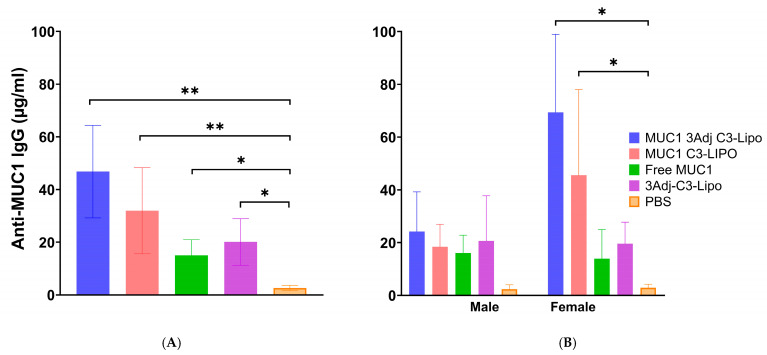
Vaccination enhances an anti-MUC1 IgG antibody response in MUC1.Tg mice. Following tumor challenge, mouse serum was collected and analyzed for an antibody response against MUC1. (**A**) All vaccine groups had a larger anti-MUC1 IgG antibody response compared to PBS controls. (**B**) Female mice vaccinated with MUC1 3Adj C3-liposomes or MUC1 C3-liposomes had significantly higher levels of anti-MUC1 IgG antibodies than female PBS controls. No significant differences were observed between male vaccine groups. Data are expressed as mean ± standard error (N = 4). (* *p*-value < 0.05; ** *p*-value < 0.01).

To evaluate MUC1-specific IgG antibody response, sera collected six weeks after the initial vaccination were analyzed by ELISA. All vaccine groups exhibited significantly higher MUC1 IgG levels compared to PBS controls (Figure 4A). Male mice showed similar IgG levels across vaccine groups (Figure 4B). In contrast, female mice immunized with MUC1 3Adj C3-liposomes or with MUC1 C3-liposomes had significantly higher IgG levels than female PBS controls (Figure 4B). Although MUC1 3Adj C3-liposome females generated a strong T-cell and IgG response, their significantly larger tumor size suggests this immune response was ineffective at controlling tumor growth. Because immune suppression could have played a role in their increased tumor growth, splenocytes from vaccinated mice were further analyzed for the presence of suppressive cell populations.

### 3.5. Vaccination with MUC1 3Adj C3-Liposomes Results in Decreased Levels of Systemic Monocytic MDSCs

**Figure 5:**
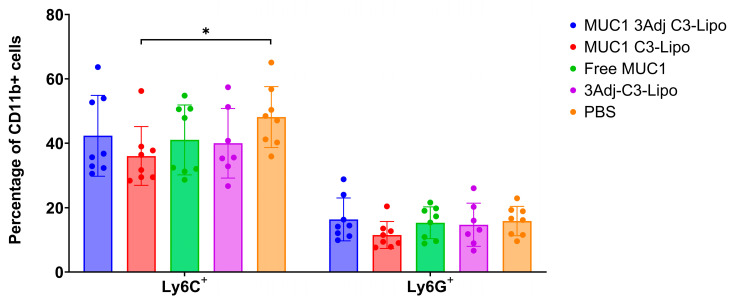
Analysis of the presence of MDSCs in spleens of vaccinated mice. After vaccination and tumor challenge, splenocytes were collected and analyzed by flow cytometry. Cells were initially gated as viable using SSC/FSC, as singlets using FSC-A/FSC-H, and then as CD45^+^CD11b^+^ myeloid cells before analysis for the presence of Ly6G and Ly6C. Mice vaccinated with MUC1 C3-liposomes had significantly fewer Ly6C^+^ myeloid cells compared to PBS control mice. No significant difference was observed in the number of Ly6G^+^ myeloid cells produced among the groups. Data are expressed as mean ± standard error (n = 8) (* *p*-value < 0.05).

Myeloid-derived suppressor cells (MDSCs) are a heterogeneous population of myeloid cells that have been shown to support tumor growth by suppressing effector immune responses and contributing to resistance to cancer treatment, particularly immunotherapies [23,24]. To understand why sex differences in tumor growth were observed, particularly in female mice vaccinated with MUC1 3Adj C3-liposomes, we evaluated the spleen cells for the presence of monocytic MDSCs (CD11b^+^Ly6C^+^) and granulocytic MDSCs (CD11b^+^Ly6G^+^) with flow cytometry. The flow cytometry gating strategy is shown in Supplementary Materials (Figure S1). Mice vaccinated with MUC1 C3-liposomes had significantly fewer monocytic MDSCs than PBS control mice (*p* = 0.02) (Figure 5). There was no significant difference in the number of granulocytic myeloid cells between the vaccination groups (Figure 5).

### 3.6. Sex Differences in MDSC Levels in Mice Vaccinated with MUC1 3Adj C3-Liposomes

**Figure 6:**
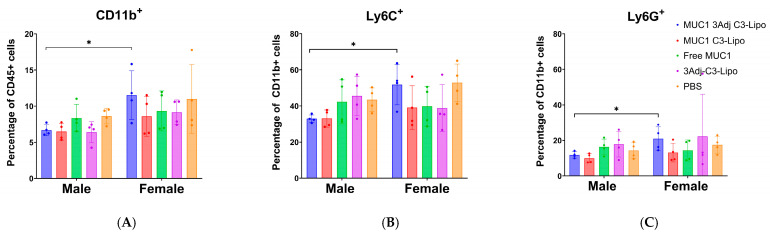
Sex differences in splenic CD11b^+^ myeloid cells. Splenic cells from each vaccine group were analyzed by flow cytometry for (**A**) CD11b^+^, (**B**) Ly6C^+^, and (**C**) Ly6G^+^ myeloid cells by sex. Female mice vaccinated with MUC1 3Adj C3-liposomes had significantly more CD11b^+^ myeloid cells than male mice. In addition, a significantly higher percentage of those CD11b^+^ myeloid cells were Ly6C^+^ and Ly6G^+^. No significant differences in myeloid cell populations by sex were observed in other vaccine groups. Cell populations are displayed as percentages of the total number of CD11b^+^ cells. Data are expressed as mean ± standard error (n = 4) (* *p*-value < 0.05).

To determine whether there were sex differences in myeloid cell populations, flow cytometry data of splenic myeloid populations were separated by sex. Interestingly, mice vaccinated with MUC1 3Adj C3-liposomes had significant sex differences. Females had a higher percentage of CD11b^+^ myeloid cells (*p* = 0.04) as well as Ly6C^+^ and Ly6G^+^ MDSCs (*p* = 0.03, 0.01 respectively) compared to male mice (Figure 6A–C). No significant differences in myeloid cell populations by sex were observed in the other vaccine groups. The increased presence of MDSCs in the female MUC1 3Adj C3-liposome group could result in systemic immune suppression and is a possible explanation for the significant difference in tumor growth between female and male mice in this vaccine group (Figure 2A). 

### 3.7. No Cytotoxicity Was Observed in Vaccinated MUC1.Tg Mice

Mouse serum was analyzed for the liver enzymes alanine transaminase (ALT) and aspartate transaminase (AST) to assess vaccine toxicity. Compared to control mice vaccinated with PBS, there were no significant differences between vaccine groups in the levels of either ALT or AST (Table 1).

## 4. Discussion

In recent years, researchers have tested MUC1-based vaccines, demonstrating that MUC1 vaccines induced long-term T-cell and antibody responses. These successes have led to several MUC1-based vaccines reaching clinical trials [7,25,26,27,28]. The aim of our study was to evaluate whether delivery of MUC1 100mer peptide and TLR agonists with C3-liposomes would effectively protect mice against a tumor challenge using MUC1-expressing tumor cells. Based on the ability of C3-liposomes to target APCs and provoke an antibody and T-cell response, we hypothesized that encapsulating TLR agonists together with the MUC1 peptide within C3-liposomes would increase MUC1 vaccine immunogenicity [16,29].

When mice were vaccinated with MUC1 C3-liposomes, which did not contain TLR agonists, female and male mice developed significantly smaller tumors than PBS controls. In contrast, vaccination with free MUC1 peptide not encapsulated in liposomes did not reduce tumor growth in either sex. These results indicate that encapsulation of the MUC1 peptides in C3-liposomes allows for the targeted delivery of MUC1 peptide to APCs and ultimately enhances the adaptive immune responses against MUC1, resulting in reduced growth of MUC1-expressing tumors [30].

Mice vaccinated with 3Adj C3-liposomes that did not contain MUC1 peptide developed significantly smaller tumors than PBS controls. This reduction in tumor growth can be attributed to female mice, as male mice did not develop tumors significantly smaller than those in PBS control mice. This sex-based difference in response to C3-liposome with TLR agonists could be explained by the fact that the immune response of females to vaccinations has been shown to be more robust than that of males [31].

When MUC1 peptide and TLR agonists were encapsulated in C3-liposomes and used to vaccinate mice, the sex differences in tumor growth were unexpected. Male mice vaccinated with MUC1 3Adj C3-liposome developed significantly smaller tumors than female mice that received the same vaccination and PBS control male mice. In addition, there was no significant difference in tumor size between the female mice vaccinated with MUC1 3Adj C3-liposomes and PBS control female mice. To determine which factors were driving tumor growth differences, we evaluated the adaptive immune response and analyzed cell populations for evidence of immune suppression.

Given the significant role of T cells, including cytotoxic T cells, in cancer immunotherapy, mice were evaluated for the presence of a MUC1-specific IFN-γ T-cell response [32,33]. Mice vaccinated with MUC1 C3-liposomes produced significantly more IFN-γ T cells compared to mice vaccinated with free MUC1 or C3-liposome-containing TLR agonists. Mice vaccinated with MUC1 peptide and TLR agonists in C3-liposomes also resulted in increased numbers of MUC1-specific T cells when compared to free MUC1 and 3Adj C3-liposomes. Interestingly, female mice vaccinated with MUC1 3Adj C3-liposomes followed a similar pattern and produced more IFN-γ-producing T cells than free MUC1 and 3Adj C3-liposomes groups, despite the significantly increased tumor growth.

Because MUC1 is found on the cell surface and often hypoglycosylated in cancer, an antibody response has the potential to lead to reduced tumor growth. Our results showed that there was a significantly higher level of MUC1 IgG antibody in mice vaccinated with all formulations than in PBS control mice. It is interesting to note that female mice vaccinated with MUC1 3Adj C3-liposomes or MUC1 C3-liposomes produced significantly more IgG antibodies than female PBS mice, but male mice of these two groups did not display the same difference [34]. Observations from prior vaccine studies suggest that this result could be due to a stronger immune response to vaccines and a more effective presentation of antigens by APCs in females [31,35]. The increased tumor growth observed in female mice vaccinated with MUC1 3Adj C3-liposomes could not be explained by a lack of adaptive immune response given the robust T-cell and antibody response in this vaccination group. It therefore seems likely that there was either a subset of cells playing a role in tumor reduction that we did not detect or that immune suppression was playing a role in preventing effective immune targeting of tumors in certain treatment groups. NK cells contribute to antitumor immunity and can mediate antibody-dependent cellular cytotoxicity (ADCC) through expression of the FcγRIIIa (CD16) receptor [36]. Likewise, T-regulatory cells (Treg) and MDSCs are immune-suppressive cell types that can greatly attenuate the adaptive immune response against a tumor [23,37]. While future studies will further evaluate NK cell and Treg populations, mice in this study were evaluated for the presence of immune-suppressive MDSCs.

MDSCs have a variety of mechanisms used to promote tumor growth, including the expression of inducible nitric oxide synthase (iNOS), as well as the arginine-metabolizing enzyme arginase I (Arg I), both of which suppress T-cell activity and the adaptive immune response against the tumor [23,24]. A MUC1 vaccine developed by Dr. Finn’s laboratory was shown to be immunogenic in 43.6% of healthy people at elevated risk for colon cancer and to induce long-term memory B-cell responses. However, in patients who did not respond to the vaccine, high levels of MDSCs were detected systemically, potentially inhibiting a robust immune response to the vaccine [22]. In our current study, flow cytometry was used to analyze and compare the populations of CD11b^+^, LY6G^+^, and LY6C^+^ myeloid cells between groups and by sex, to determine the presence of MDSCs [38]. PBS controls produced significantly more Ly6C^+^ MDSCs than mice vaccinated with MUC1 C3-liposomes, whereas there was no significant difference in the percentage of Ly6G^+^ MDSCs. This expression pattern indicates that monocytic MDSCs may play a larger role in immune suppression than granulocytic MDSCs [39]. In addition, there was a significant difference in the percentage of CD11b^+^, Ly6G^+^, and Ly6C^+^ cells between male and female mice vaccinated with MUC1 3Adj C3-liposomes, with female mice having a higher percentage of MDSCs compared to male mice. The high number of MDSCs detected in the female mice might explain why their tumors grew significantly more quickly than those in the male mice.

To assess the safety of our vaccine, we evaluated serum alanine aminotransferase (ALT) and aspartate aminotransferase (AST) as the primary indicators of liver toxicity and injury [40]. All vaccinated mice had low levels of ALT/AST, with no significant difference between groups or sexes. Our findings are consistent with those from our previous study [21].

## 5. Conclusions

Our study demonstrates that encapsulating MUC1 in C3-liposomes generates a cancer vaccine capable of eliciting an adaptive T-cell and B-cell immune response, leading to reduced tumor growth [41]. Previous research has shown that incorporating TLR agonists into C3-liposome formulations enhances the immune response [17,21]. However, in this tumor challenge model, the addition of TLR agonists led to a striking sex-based difference in tumor growth. While MUC1 C3-liposomes containing TLR agonists significantly reduced tumor growth in male mice, the vaccination had no effect on tumor growth in females. Despite the vaccine eliciting a strong adaptive immune response in male and female mice, in females, it promoted MDSC expansion, potentially suppressing anti-tumor efficacy. These results highlight the complexity of sex differences in immune response and the potential impact of MDSCs on vaccine efficacy, as first observed by Dr. Finn et al. [22]. It is possible that a different adjuvant combination could maintain an immune stimulating response while attenuating the recruitment of myeloid cells and their polarization to an immune suppressive phenotype. Our findings emphasize the need for careful consideration when incorporating TLR agonists into vaccine formulations and highlight the importance of further research into sex differences in immune responses to cancer vaccines and immunotherapy.

## Figures and Tables

**Table 1:** Liver enzyme activity in mouse serum samples.

Vaccination Group	ALT * (U/L)	AST † (U/L)
MUC1 3Adj C3-liposomes	37.25 ± 11.37	23.53 ± 5.10
MC1 C3-liposomes	56.06 ± 26.82	15.34 ± 5.21
Free MUC1	26.05 ± 8.19	22.17 ± 4.28
3Adj C3-liposomes	67.20 ± 31.23	20.08 ± 5.74
PBS	39.74 ± 12.90	10.12 ± 6.34

Data are presented as mean ± SD (n = 3). * Alanine transaminase; † aspartate transaminase.

## Data Availability

The original contributions presented in this study are included in the article/Supplementary Material. Further inquiries can be directed to the corresponding author.

## Supplementary Materials

The following supporting information can be downloaded at: https://www.mdpi.com/article/10.3390/pharmaceutics17040468/s1, Figure S1. Flow Cytometry Gating Strategy: Live cells were selected by forward scatter/side scatter. Singlets were identified by gating forward scatter-A (area)/forward scatter-H (height). Myeloid cells were identified as CD45^+^CD11b^+^ and these were further broken down into Ly6G^+^ and Ly6C^+^ populations. Table S1. The *p*-values for all significant data reported in the manuscript are shown here.
