# Supplementary material for: C3-Liposome Delivery of MUC1 Peptide and TLR Agonists Enhances Adaptive Immunity and Results in Sex-Based Tumor Growth Differences
Source: Pharmaceutics. 2025 Apr 3;17(4):468. doi: 10.3390/pharmaceutics17040468 (PMC12030583; doi:10.3390/pharmaceutics17040468)
Supplement: Supplementary file 1 [file pharmaceutics-17-00468-s001.zip › pharmaceutics-3516147-supplementary.pdf]

Supplementary Materials:

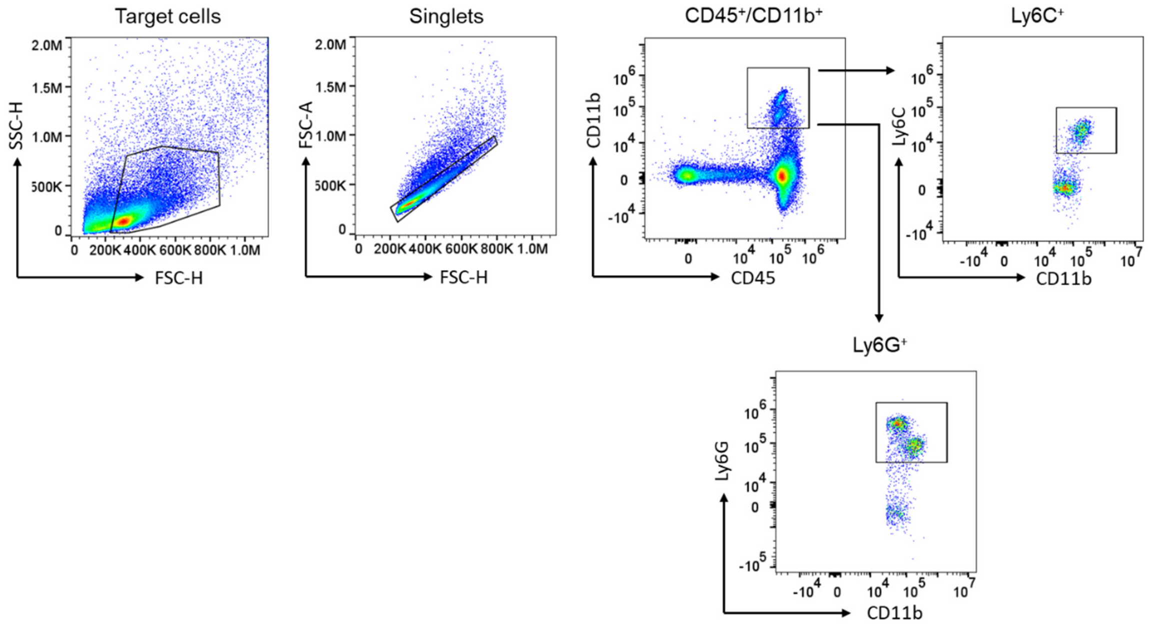

Figure S1. Flow Cytometry Gating Strategy: Live cells were selected by forward scatter/side scatter. Singlets were identified by gating forward scatter-A (area)/forward scatter-H (height). Myeloid cells were identified as $CD45^{+}CD11b^{+}$ and these were further broken down into $Ly6G^{+}$ and $Ly6C^{+}$ populations.

Table S1. The p-values for all significant data reported in the manuscript are shown here.

**Figure #**	**Group 1**	**Group 2**	**p value**
Figure 1	PBS	MUC1 C3-Lipo	0.006
Figure 1	PBS	3Adj-C3-Lipo	0.004
Figure 2A	F MUC1 3Adj C3-Lipo	M MUC1 3Adj C3-Lipo	0.003
Figure 2A	M PBS	M MUC1 3Adj C3-Lipo	0.008
Figure 2B	M PBS	M MUC1 C3-Lipo	0.01
Figure 2B	F MUC1 C3-Lipo	F PBS	0.04
Figure 2C	F PBS	F 3Adj C3-Lipo	0.003
Figure 3A	MUC1 3Adj C3-Lipo	3Adj C3-Lipo	0.01
Figure 3A	MUC1 3Adj C3-Lipo	Free MUC1	0.01
Figure 3A	MUC1 C3-Lipo	Free MUC1	0.02
Figure 3A	MUC1 C3-Lipo	3Adj C3-Lipo	0.02
Figure 3B	F MUC1 3Adj C3-Lipo	F Free MUC1	0.04
Figure 3B	F MUC1 3Adj C3-Lipo	F 3Adj C3-Lipo	0.04
Figure 4	MUC1 3Adj C3-Lipo	PBS	0.001
Figure 4	MUC1 C3-Lipo	PBS	0.001
Figure 4	Free MUC1	PBS	0.04
Figure 4	3Adj C3-Lipo	PBS	0.01
Figure 4	F MUC1 3Adj C3-Lipo	PBS	0.02
Figure 4	F MUC1 C3-Lipo	PBS	0.02
Figure 5	MUC1 C3-Lipo	PBS	0.02
Figure 6	M CD11b	F CD11b	0.04
Figure 6	M Ly6C	F Ly6C	0.03
Figure 6	M Ly6G	F Ly6G	0.01
